# Development of a personalized diagnostic model for kidney stone disease tailored to acute care by integrating large clinical, demographics and laboratory data: the diagnostic acute care algorithm - kidney stones (DACA-KS)

**DOI:** 10.1186/s12911-018-0652-4

**Published:** 2018-08-17

**Authors:** Zhaoyi Chen, Victoria Y. Bird, Rupam Ruchi, Mark S. Segal, Jiang Bian, Saeed R. Khan, Marie-Carmelle Elie, Mattia Prosperi

**Affiliations:** 1 Department of Epidemiology, College of Public Health and Health Professions & College of Medicine, University of Florida, 2004 Mowry Road, PO Box 100231, Gainesville, Florida 32610-0231 USA; 2 Department of Urology, University of Florida, Gainesville, Florida USA; 3 Division of Nephrology, Hypertension, & Renal Transplantation, University of Florida, Gainesville, Florida USA; 4 Department of Health Outcomes and Biomedical Informatics, University of Florida, Gainesville, Florida USA; 5 Department of Pathology, Immunology, and Laboratory Medicine, Gainesville, Florida USA; 6 Department of Emergency Medicine, University of Florida, Gainesville, Florida USA

**Keywords:** Diagnostic algorithm, Kidney stones, Big data analysis

## Abstract

**Background:**

Kidney stone (KS) disease has high, increasing prevalence in the United States and poses a massive economic burden. Diagnostics algorithms of KS only use a few variables with a limited sensitivity and specificity. In this study, we tested a big data approach to infer and validate a ‘multi-domain’ personalized diagnostic acute care algorithm for KS (DACA-KS), merging demographic, vital signs, clinical, and laboratory information.

**Methods:**

We utilized a large, single-center database of patients admitted to acute care units in a large tertiary care hospital. Patients diagnosed with KS were compared to groups of patients with acute abdominal/flank/groin pain, genitourinary diseases, and other conditions. We analyzed multiple information domains (several thousands of variables) using a collection of statistical and machine learning models with feature selectors. We compared sensitivity, specificity and area under the receiver operating characteristic (AUROC) of our approach with the STONE score, using cross-validation.

**Results:**

Thirty eight thousand five hundred and ninety-seven distinct adult patients were admitted to critical care between 2001 and 2012, of which 217 were diagnosed with KS, and 7446 with acute pain (non-KS). The multi-domain approach using logistic regression yielded an AUROC of 0.86 and a sensitivity/specificity of 0.81/0.82 in cross-validation. Increase in performance was obtained by fitting a super-learner, at the price of lower interpretability. We discussed in detail comorbidity and lab marker variables independently associated with KS (e.g. blood chloride, candidiasis, sleep disorders).

**Conclusions:**

Although external validation is warranted, DACA-KS could be integrated into electronic health systems; the algorithm has the potential used as an effective tool to help nurses and healthcare personnel during triage or clinicians making a diagnosis, streamlining patients’ management in acute care.

## Background

Kidney stone (KS) disease prevalence has increased in the United States from 5.2% (6.3% males and 4.1% females) in 1994 to 8.8% (10.6% males and 7.1% females) in 2012 [1]. Since it is one of the costliest urologic diseases in the United States, an increase in prevalence poses a huge economic burden on society. The cost of diagnosis, treatment and prevention of KS disease in 2007 was estimated to be ~$4 billion and, due to population growth alone, is projected to increase by more than $780 million by 2030 [2, 3]. The presence of KS also places the individuals at increased risk of development of chronic kidney disease. In a prospective cohort study, those who had KS was associated with a 50–67% higher risk of developing chronic kidney disease as compared to those who did not have, KS group also had twice the risk of developing end-stage renal disease [4].

The emergency department (ED) is a common place where patient with KS are evaluated and diagnosed. During the past two decades, a significant increase in ED visits with stone-related symptoms has been observed [5], with over 1.3 million individuals per year presenting to the ED with KS in the United States. The clinical presentation to the ED with KS commonly involves acute back, flank or groin pain, nausea, vomiting and sometimes blood in urine. The workup may include initial lab tests such as complete blood count with differential, comprehensive metabolic panel, and urine analysis; but often these tests are not promptly measured or are inappropriately interpreted [5].

A cross-sectional analysis of the 2007–2010 National Health and Nutrition Examination Survey (NHANES) dataset suggests that obesity, diabetes, and gout all have a significant positive association with kidney stone history [1]. Results from the Nurses’ Health Study, a large population-based longitudinal study (years 2001–2012) demonstrated that high body-mass index (BMI), cholelithiasis, diabetes and specific dietary factors are associated with a higher risk of KS formation in females [6]. In 2014, a clinical prediction score -named STONE- was derived and validated in retrospective and prospective cohorts [7]. The STONE score includes five variables: male sex, short duration of pain, non-black race, presence of nausea or vomiting, and microscopic hematuria. The STONE score was also externally validated and showed good validity in patients with flank pain [8]. An updated STONE-PLUS score, augmented by point-of-care limited ultrasonography assessing hydronephrosis, was recently released and tested prospectively on an ED population sample, with only a moderate improvement in risk stratification [9]. As KS disease is multifactorial in nature, we hypothesized that an approach incorporating laboratory data and additional clinical characteristics would dramatically improve a KS diagnostic model, leading to earlier diagnosis and a better understanding of its complex etiology. In addition, this approach could reduce the number of unnecessary radiographic testing i.e. CT scans, in the acute care setting.

In this study, we tested a big data approach, merging demographic, vital signs, clinical, and laboratory information, to infer and validate a ‘multi-domain’ personalized diagnostic score for KS. We utilized a large, single-center database of patients admitted to ED and other intensive/acute care units in a large tertiary care hospital (over 58,000 admissions with majority admitted through ED). We analyzed the information domains individually (e.g. only comorbidities, or only lab tests), together, and compared our approach with the STONE score. A number of statistical and machine learning models were fit and compared to optimize performance. Using this multi-domain integration approach our goal was to significantly improve the sensitivity and specificity of KS diagnosis in acute settings.

## Methods

### Study population

The study population comprised individuals admitted to critical care units at the Beth Israel Deaconess Medical Center in Boston, Massachusetts, United States, between 2001 and 2012. Data are stored electronically in the Medical Information Mart for Intensive Care (MIMIC-III) database, which is available to the public upon request, upon Collaborative Institutional Training Initiative (CITI) training, and license agreement for full download and research [10]. MIMIC-III includes information on: demographics; clinical diagnoses and procedures encoded with the International Classification of Diseases ver. 9 (ICD-9) ontology; vital sign measurements made at the bedside (~ 1 data point per hour); laboratory test results; medications; caregiver notes; imaging reports; mortality (both in- and out-of-hospital).

This is a secondary data analysis. We used the MIMIC-III ver. 1.4, released on September 2nd, 2016. Our study included patients aged 18 years and older, divided into four groups based on the ICD-9 diagnoses during hospitalization: (a) KS cases (ICD-9592, including sub-codes 592.0, 592.1, 592.9); (b) patients diagnosed with genitourinary diseases (GUD) except KS (any ICD-9 code in the intervals 580–591 or 593–599), e.g. patients with nephritis, nephrotic syndrome, nephrosis; (c) patients admitted to acute care with other conditions (OTH) who did not have any KS or GUD diagnosed (any ICD-9 code not including 580–599) to represent a general patient population; (d) patients admitted with acute localized pain (ALP) of abdominal (ICD-9 code: 789.0), back (ICD-9 code: 724.2), flank, or groin (identified through patients’ electronic chart record). In addition to ICD-9 codes, we also examined recorded charted events on ALP from the dataset. Patients with both KS and GUD codes were put into the KS group. Each patient was associated to a covariate vector of demographic info, vital signs, clinical diagnoses, procedures, medicaments, and laboratory tests performed during hospitalization.

### Statistical analysis

Descriptive analysis was used to assess demographic characteristics (e.g. gender, age, insurance status, and religion), vital signs (e.g. BMI, blood pressure), laboratory tests (e.g. creatinine), and distribution of ICD-9 diagnoses at admission and during hospitalization. We also calculated the Charlson Comorbidity Index (CCI) using Deyo’s algorithm [11], and the estimated glomerular filtration rate (eGFR) using the CKD-EPI (Chronic Kidney Disease Epidemiology Collaboration) equation equation [12].

Due to a low frequency of KS, we included only ICD-9 diagnostic codes that were occurred in less than 5 counts of the KS group, and lab tests that performed in at least 50% of the KS formers. Missing values were imputed via population median/mode. Univariate analysis was conducted to assess differences between KS and GUD/OTH/ALP groups on demographics, ICD-9 diagnoses, and lab tests, using Student’s t-test, Wilcoxon rank test, or chi-square test, where appropriate. Significance *p*-values were adjusted using False Discover Rate (FDR) correction [13].

In order to infer a KS diagnostic score, we fitted a collection of multivariable logistic regression models with the GUD, OTH or ALP as negative examples, using different input covariate domains. Specifically, we evaluated seven models: (a) demographic variables and vital signs (including blood pressure, heart rate and body temperature) (b) CCI, plus demographic variables; (c) eGFR alone; (d) ICD-9 diagnosis (top-25 as selected by the univariate filter, i.e. the top-25 variables that were differently distributed between KS and other groups), plus demographic variables; (e) laboratory tests (top-25 as selected by the univariate filter), plus demographic variables; (f) ICD-9 diagnosis and laboratory tests (top-50 as selected by the univariate filter), plus all other variables included in models (a) to (e); (g) stepwise (forward-backward) selection of model (f); (h) STONE model. Note that ICD-9 codes used to define the GUD were not used as input covariates to any of the models, except for the STONE model where hematuria (ICD-9 code 599.7) is a covariate. Also, the duration of pain to presentation in the STONE score could not be precisely ascertained from our data; we used ICD-9 codes in the 338 s family plus codes 780.96 and 789.0, excluding chronic pain entries, using a weight of 2 (the STONE score a < 6 h pain is weighted 3 and 6–24 h pain is weighted 1, but duration of pain was not available in our data set). In addition to ICD-9 codes, we also used charted events to identify pain events. For nausea/vomiting we used ICD-9787.0 codes. In a sensitivity analysis, we also evaluated the contribution of GUD codes to overall performance of models (d) to (g).

Model comparison, evaluation, and selection were carried out using a 10-fold cross-validation framework [14], comparing performance index (see below) distributions from the repeated sampling folds using Bengio and Nadeau’s correction to the Student’s t-test [15].however, th.

In addition to logistic regression, we also fit a number of machine learning techniques on the full variable set as in model (f). In details: (i) a *decision tree* by means of the C4.5 algorithms [16]; (ii) *LogitBoost* algorithm in conjunction to logistic regression [17]; (iii) a *random forest* (optimizing number of trees up to 1000) [18]; (iv) a *super learner* stacking all the above methods plus a single-rule linear model, internally optimized via 5-fold cross-validation [19]. Given the high class imbalance, in addition to the standard model fit, we also used the synthetic minority over-sampling technique (SMOTE) internally to the cross-validation [20]. The univariate feature selection for these machine learning algorithms was done internally within the cross-validation setting.

The performance and discriminative ability of models was assessed using sensitivity (true positive rate), specificity (true negative rate), and the area under the receiver operating characteristic (AUROC), which is the expectation that a uniformly drawn random positive case is ranked before a uniformly drawn random negative (an area of 100% represents a perfect test; an area of 50% represents a worthless test) [21]. The optimal sensitivity/specificity cutoff was chosen based on the maximal of the Youden’s J statistic [22]. All statistical analyses were conducted using SAS software ver. 9.4 (SAS Institute Inc., Cary, NC, USA) and Weka ver. 3.9 [23].

## Results

There were 38,597 distinct adult patients (> 18-year-old) in the MIMIC-III database admitted to critical care units between June 2001 to October 2012 (90% from emergency room admission, 8% elective surgery, and 2% urgent care services), of which 217 were diagnosed with KS, 14,391 with GUD, 23,931 as OTH who did not have any GUD nor KS, and 7446 as ALP with abdominal, back, flank, groin pain.

**Fig. 1:**
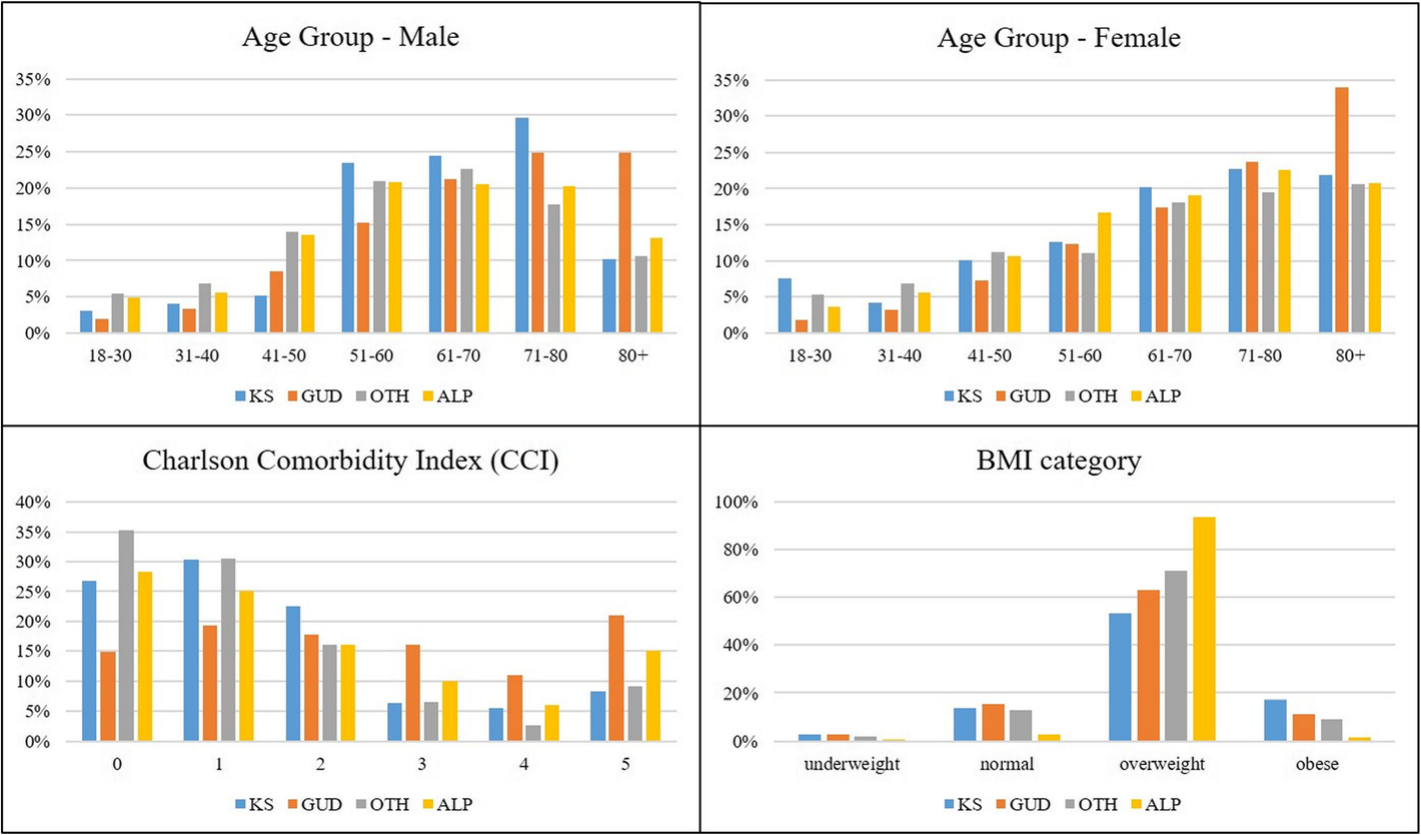
Distributions of age categories by gender, CCI and BMI in KS, GUD, OTH and ALP groups

Table 1 summarizes population characteristics among the three groups. There was an excess of females in the KS group as compared to other three groups (45.2% vs. 54.3%, 58.1% and 52.4%, respectively, *p* <  0.05). Most sample population were admitted through emergency or urgent (84.2%). The distribution of race was similar between KS and GUD, but comparing to OTH and ALP, KS had a higher proportion of white (76.5% vs. 71.1% and 72.7%) and black African American (10.6% vs 6.0% and 7.4%, *p* = 0.008). The median eGFR in KS was 65.3, lower than in OTH (93.1, *p* = 0.0013) and ALP (77.3, *p* <  0.0001), but higher than GUD (49.3, p <  0.0001). The median (IQR) STONE score in KS formers was 4, higher than in GUD (2, p <  0.0001) or in OTH (2, *p* < 0.0001), but not different from ALP (4, *p* = 0.46). Figure 1 shows the comparison of the distributions of age categories by gender, CCI and BMI in the three groups of KS, GUD and OTH. The highest rates of KS were seen in the age group 71–80 for both males (30%) and females (23%), and the rates of KS increased significantly after 50 years-of-age in males, while in females a steady increase was observed after 30 years-of-age with a leveling off after 70 years. As for BMI, KS had the highest overall distribution (median 29.1) among all four groups (median of GUD, OTH and ALP: 27.5, 27.2, 27.0), it also had the highest proportion of obese (17% vs 11% in GUD, 9% in OTH and 2% in ALP, all *p*-values < 0.05).

**Table 1:** Characteristics of the study population (*n* = 38,597), stratified by outcome group

	kidney stones (KS)	other genitourinary diseases (GUD)		other conditions (OTH)		acute localized pain (ALP)	
	% (N)	% (N)	*p*-value	% (N)	*p*-value	% (N)	*p*-value
Total	217	14,391		23,931		7446	
Gender							
Male	45.2% (98)	54.3% (7816)	0.0072	58.1% (13895)	0.0001	52.4% (3902)	0.04
Ethnicity							
White	76.5% (166)	72.0% (10359)	0.34	71.1% (17007)	0.0008	72.7% (5413)	0.04
Black	10.6% (23)	10.4% (1493)		6.0% (1445)		7.4% (549)	
Hispanic	3.2% (7)	2.9% (416)		3.5% (833)		3.1% (231)	
Asian	1.4% (3)	2.5% (358)		2.3% (555)		1.7% (124)	
Other	8.3% (18)	12.2% (1765)		17.1% (4091)		15.2% (1129)	
Insurance							
Medicare/Medicaid/Government	67.3% (146)	77.6% (11164)	0.0002	57.8% (13837)	0.0094	64.7% (4814)	0.48
Self pay/Private	32.3% (70)	21.7% (3130)		40.4% (9671)		34.4% (2560)	
Missing	0.5% (1)	0.7% (97)		1.8% (423)		1.0% (72)	
Admission type							
Elective	9.2% (20)	8.2% (1176)	0.92	20.2% (4841)	< 0.0001	16.0% (1193)	0.01
Emergency	89.4% (194)	89.6% (12895)		76.9% (18406)		80.7% (6005)	
Urgent	1.4% (3)	2.2% (320)		2.9% (684)		3.3% (248)	
	Median (IQR)	Median (IQR)		Median (IQR)		Median (IQR)	
Age	67 (56–77)	72 (59–82)	< 0.0001	62 (50–75)	0.0049	64 (51–77)	0.083
BMI	29.1 (28.3–30.5)	27.5 (26.9–27.6)	0.15	27.2 (27.2–27.2)	0.0023	27.0 (26.9–27.1)	< 0.0001
Charlson Index	1 (0–2)	2 (1–4)	< 0.0001	1 (0–2)	0.74	1 (0–3)	0.12
eGFR	50.8 (33.4, 81.9)	38.9 (22.5, 62.3)	0.0013	82.5 (59.1, 98.7)	< 0.0001	65.1 (37.3, 93.9)	< 0.0001
STONE	4 (2–4)	2 (2–4)	< 0.0001	2 (2–4)	< 0.0001	4 (2–4)	0.46

**Fig. 2:**
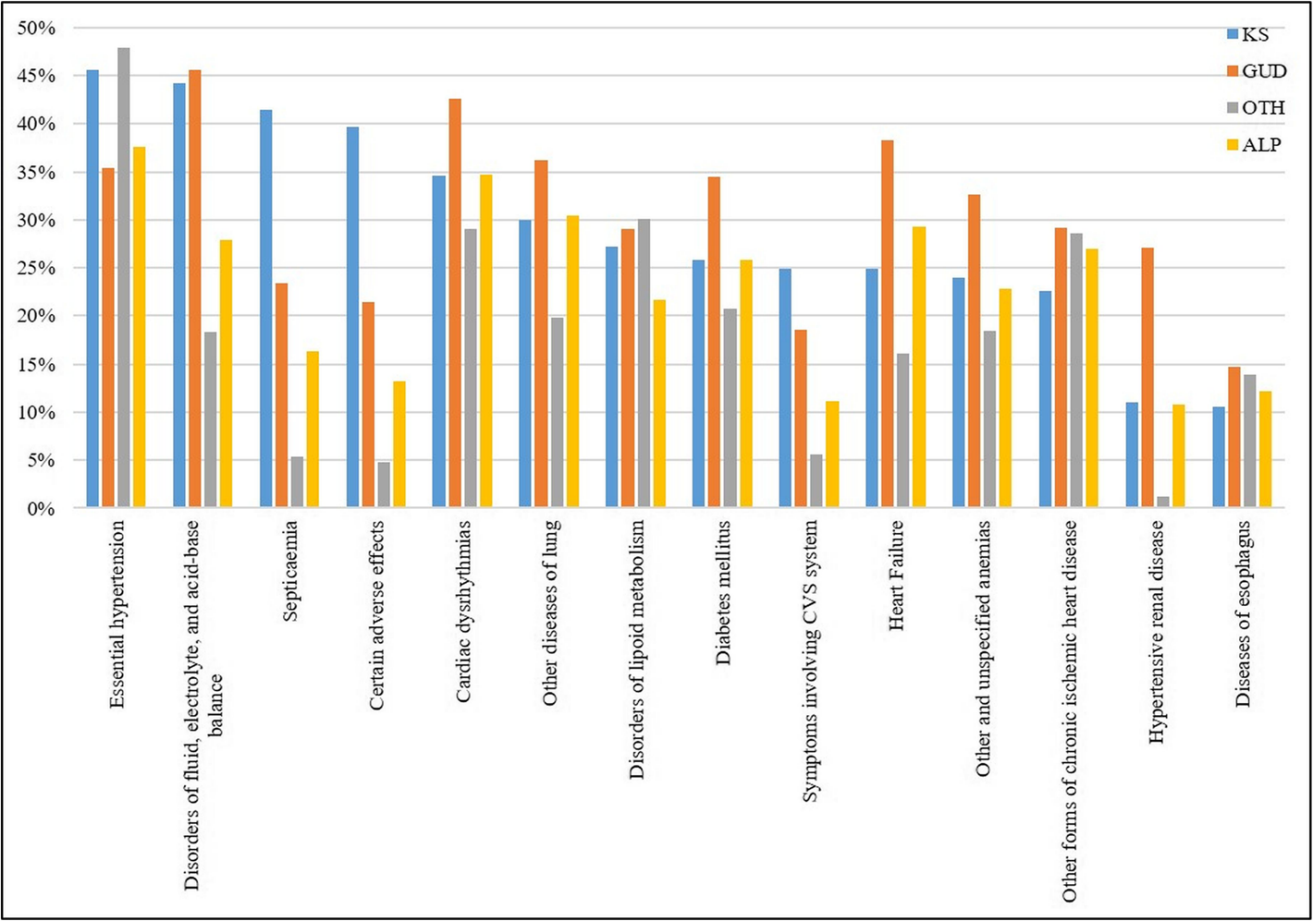
Prevalence of the top-10 most frequent ICD-9 diagnoses in KS, GUD, OTH and ALP groups

Figure 2 shows the most frequent ICD-9 diagnoses in all four groups of KS, GUD, OTH and ALP, collating the top-10 frequencies of each group. Essential hypertension (45.8%), disorders of fluid, electrolyte, and acid-base balance (44%), and septicemia (41.7%) were most frequently diagnosed conditions among KS patients. Some of these high frequency comorbidities also had different distribution in KS compared to other groups. For example, rates of septicemia and certain adverse effects (including anaphylaxis, unspecified medication adverse effects, unspecified allergy, etc.) in KS were higher than in GUD, OTH or ALP (18%, 36% and 23% higher respectively). The proportion of essential hypertension was 10% higher in KS than GUD or ALP but was similar to the rate in OTH; heart failure and hypertensive renal disease had much lower rates (14% and 16% less respectively) in KS than in GUD, but the rates were higher in KSF comparing to OTH (8% and 10% higher).

When looking at the STONE variables, we found that hematuria was positively associated with KS (7.4% vs. 4.6% in GUD, *p* = 0.051, and vs. 1.1% in OTH, *p* < 0.0001, and vs. 1.5% in ALP, *p* < 0.0001); 98.6% of KS formers had experienced pain while 53.1% of GUD and 57.6% of OTH had pain events (both p < 0.0001); 0.92% of KS formers had vomiting and 0.46% had nausea recorded, and the percentages of vomiting and nausea in KS were slightly higher than in other three groups. Hydronephrosis (variable from STONE-PLUS) was also positively associated with KS (35.94% vs. 1.54% in GUD, *p* < 0.0001 and vs. 0% in OTH, *p* < 0.0001).

Next, we performed univariate analysis of ICD-9 diagnosis and lab tests comparing KS with GUD/OTH/ALP. A total of 940 distinct three-letter ICD-9 codes were identified in the whole study population; after code filtering based on low frequency (< 5 cases in KS), 83 variables remained. For laboratory tests, a total of 754 entries were found, further condensed to 637 by manual inspection of physicians, and reduced to 69 after frequency filtering. The frequencies of missing values of these included lab tests ranges from 0 to 45%, 66.0% and 45.2% in GUD, OTH and ALP respectively, with the majority of them have less than 50% of missing.

Table 2 shows frequencies of the top ICD-9 diagnosis identified through univariate analysis, selecting those with an FDR-adjusted *p*-value below 0.1 (up to the top-25). Overall, 7 ICD-9 were differentially distributed between KS and GUD at the 5% FDR level, while 25 of them were found different between KS and OTH or ALP at the same significance level. Out of the 69 lab tests performed in more than half of KS patients, 43, 50, and 25 showed a significant (5% FDR level) mean or distribution location shift between KS vs. GUD, KSF vs. OTH, and KSF vs. ALP, respectively. The top-25 lab tests rank is shown in Table 3.

**Table 2:** Top-ranked ICD-9 diagnoses differentially associated with KS vs. GUD / OTH / ALP

ICD-9 code	Condition	frequency in kidney stones (KS)	other genitourinary diseases (GUD)		other conditions (OTH)		acute localized pain (ALP)	
			frequency	*p*-value*	frequency	*p*-value*	frequency	*p*-value*
401	Essential hypertension	45.6% (99)	35.4% (5101)	0.02	48.5% (11447)	0.63	37.6% (2797)	0.0224
276	Disorders of fluid, electrolyte, and acid-base balance	43.8% (95)	45.6% (6565)	0.78	18.3% (4375)	< 0.0001	27.9% (2047)	< 0.0001
38	Septicaemia	41.5% (90)	23.4% (3361)	< 0.0001	5.4% (1297)	< 0.0001	16.3% (1216)	< 0.0001
995	Certain adverse effects	39.6% (86)	21.4% (3078)	< 0.0001	4.8% (1159)	< 0.0001	13.2% (983)	< 0.0001
785	Symptoms involving cardiovascular system	24.9% (54)	18.5% (2664)	0.09	5.7% (1353)	< 0.0001	11.1% (827)	< 0.0001
428	Heart failure	24.9% (54)	38.3% (5508)	0.00	16.2% (3867)	0.002	29.3% (2185)	0.1945
41	Other bacteria infections	21.2% (46)	16.3% (2350)	0.14	3.1% (752)	< 0.0001	7.5% (559)	< 0.0001
287	Purpura and other hemorrhagic conditions	12.9% (28)	11.7% (1680)	0.76	5.1% (1211)	< 0.0001	7.9% (589)	0.0293
790	Nonspecific findings on examination of blood	12.0% (26)	9.8% (1413)	0.54	5.2% (1243)	< 0.0001	6.0% (449)	0.0019
403	Hypertensive renal disease	11.1% (24)	27.0% (3892)	< 0.0001	1.2% (278)	< 0.0001	10.8% (805)	0.9642
278	Obesity and other hyperalimentation	10.1% (22)	6.0% (863)	0.08	4.7% (1121)	0.001	4.2% (315)	0.0003
311	Depressive disorder, not elsewhere classified	10.1% (22)	7.7% (1104)	0.77	5.9% (1403)	0.01	4.7% (351)	0.0015
300	Neurotic disorders	9.2% (20)	5.68% (817)	0.08	5.71% (1366)	0.03	4.0% (295)	0.0003
327	Sleep disorders	9.2% (20)	5.4% (778)	0.09	3.5% (843)	< 0.0001	2.9% (212)	< 0.0001
112	Candidiasis	8.8% (19)	4.3% (619)	0.02	2.0% (479)	< 0.0001	4.0% (298)	0.0023
416	Chronic pulmonary heart disease	7.8% (17)	6.9% (989)	0.77	3.4% (809)	0.001	4.3% (317)	0.0365
799	Decreased libido and other ill-defined conditions	7.4% (16)	4.7% (668)	0.19	2.4% (569)	< 0.0001	2.5% (186)	< 0.0001
788	Symptoms involving urinary system	7.4% (16)	5.7% (820)	0.77	3.0% (712)	0.001	3.1% (229)	0.0019
288	Diseases of white blood cells	6.0% (13)	4.7% (674)	0.77	2.6% (629)	0.001	2.0% (148)	0.0004
574	Cholelithiasis	5.5% (12)	2.9% (419)	0.11	1.6% (392)	< 0.0001	3.1% (230)	0.0558
570	Acute and subacute necrosis of liver	4.1% (9)	5.0% (717)	0.76	0.8% (184)	< 0.0001	3.1% (228)	0.4404
345	Epilepsy	3.7% (8)	3.4% (491)	0.87	2.9% (688)	0.48	1.5% (111)	0.0345
346	Migraine	2.8% (6)	0.79% (113)	0.02	1.32% (316)	0.11	0.8% (57)	0.0052

* first 25 ICD-9 codes or those with a false discovery rate-adjusted p-value< 0.05 are shown

**Table 3:** Top-ranked laboratory tests differentially associated with KS vs. GUD / OTH / ALP

Lab test item	kidney stones (KS)		other genitourinary diseases (GUD)			other conditions (OTH)			acute localized pain (ALP)		
	mean	Median (IQR)	Mean	Median (IQR)	*p*-value*	Mean	Median (IQR)	*p*-value*	Mean	Median (IQR)	*p*-value*
*Urine*											
White Blood Cells (WBC) (#/hpf)	5.5	4.9 (3.1, 6.2)	3.0	2.2 (1.7, 2.9)	< 0.0001	1.5	1.5 (1.5, 1.5)	< 0.0001	2.1	1.2 (1.2, 2.2)	< 0.0001
Red Blood Cells (RBC) (#hpf)	5.8	4.5 (3.3, 6.2)	3.4	2.4 (1.7, 3.3)	< 0.0001	1.9	1.4 (1.4, 1.4)	< 0.0001	2.7	1.4 (1.4, 2.4)	< 0.0001
Creatinine (mg/dL)	80.0	73.0 (73.0, 73.0)	84.7	78.0 (70.5, 86.0)	0.03	77.9	75.0 (75.0, 75.0)	0.03	81.9	75.0 (75.0, 78.0)	0.03
Protein (mg/dL)	4.0	4.0 (3.4, 4.2)	3.9	3.8 (3.4, 4.0)	0.03	3.5	3.4 (3.4, 3.4)	< 0.0001	3.7	3.4 (3.4, 3.8)	< 0.0001
pH	6.0	6.0 (5.5, 6.5)	5.8	5.7 (5.2, 6.2)	< 0.0001	6.0	6.0 (5.5, 6.5)	0.86	6.0	6.0 (5.4, 6.5)	0.17
*Blood*											
Bands	4.5	3.0 (2.0, 3.8)	2.5	1.3 (1.0, 1.9)	< 0.0001	1.5	0.8 (0.8, 0.8)	< 0.0001	2.3	0.8 (0.5, 2.0)	< 0.0001
Potassium (mEq/L)	4.1	4.0 (3.9, 4.1)	4.3	4.2 (4.0, 4.4)	< 0.0001	4.1	4.1 (3.9, 4.2)	0.09	4.2	4.1 (3.9, 4.3)	< 0.0001
Lipase (U/L)	67.1	28.0 (26.0, 33.0)	62.6	35.0 (30.0, 42.0)	0.03	44.9	31.0 (31.0, 31.0)	0.05	65.4	31.0 (27.0, 45.0)	0.03
Magnesium (mEq/L)	2.0	2.0 (1.8, 2.1)	2.1	2.0 (1.9, 2.2)	< 0.0001	2.0	2.0 (1.9, 2.1)	0.03	2.0	2.0 (1.9, 2.1)	0.01
Glucose (mg/dl)	146.0	137.3 (137.3, 137.3)	143.6	134.0 (127.2, 141.0)	0.03	139.5	133.3 (127.6, 139.7)	0.03	141.0	133.3 (124.2, 145.3)	0.03
Creatine Kinase, MB Isoenzyme (ng/mL)	8.5	4.4 (4.0, 5.0)	0.0	5 (4.0, 6.8)	0.03	11.5	5.0 (5.0, 5.0)	0.32	11.9	5 (4.7, 6)	0.03
Red Cell Distribution Width (RDW) (%)	14.9	14.5 (13.7, 15.7)	15.7	15.3 (14.2, 16.7)	< 0.0001	14.5	14.1 (13.4, 15.2)	0.001	15.3	15.0 (13.92, 16.3)	0.005
Total CO_2_ (mEq/L)	24.0	24.0 (22.0, 25.5)	24.8	24.8 (22.7, 26.9)	0.03	25.9	25.8 (24.7, 27.0)	< 0.0001	25.7	25.8 (23.8, 27.4)	< 0.0001
Red Blood Cells (cells/mcL)	3.7	3.6 (3.3, 4.0)	3.5	3.4 (3.2, 3.8)	< 0.0001	3.7	3.7 (3.3, 4.1)	0.09	3.6	3.5 (3.3, 3.8)	0.01
Chloride (mEq/L)	102.6	102.4 (101.3, 103.7)	101.9	102.0 (100.5, 103.3)	0.001	101.9	102.0 (100.5, 103.0)	< 0.0001	101.8	101.9 (100.7, 103.0)	< 0.0001
Urea Nitrogen (mg/dL)	25.7	20.8 (14.1, 30.7)	34.5	29.5 (19.5, 44.4)	< 0.0001	17.8	15.8 (12.0, 21.0)	< 0.0001	25.7	20.1 (13.8, 32.3)	0.95
Creatinine (mg/dL)	1.4	1.1 (0.8, 1.6)	1.9	1.4 (1.0, 2.1)	0.003	0.9	0.8 (0.7, 1.0)	< 0.0001	1.3	0.9 (0.7, 1.4)	0.80
Albumin (g/dL)	3.3	3.2 (2.9, 3.6)	3.1	3.1 (2.8, 3.5)	0.002	3.5	3.5 (3.3, 3.7)	< 0.0001	3.2	3.3 (2.8, 2.6)	0.31
Phosphate (mg/dL)	3.3	3.2 (2.8, 3.7)	3.7	3.5 (3.1, 4.1)	< 0.0001	3.3	3.3 (2.9, 3.6)	0.60	3.5	3.4 (3.0, 3.9)	< 0.0001
Oxygen Saturation (mm Hg)	90.1	92.0 (92.0, 92.0)	90.3	92.1 (90.4, 93.8)	0.78	93.4	95.3 (95.3, 95.3)	< 0.0001	91.5	95.0 (90.3, 95.6)	0.02
Base Excess (mEq)	−1.6	−1.4 (−2.7, −0.3)	−1.0	−0.6 (−2.5, 0.9)	0.15	0.1	0.1 (−0.6, 1.0)	< 0.0001	−0.2	0.1 (−1.6, 1.4)	< 0.0001
pH	7.4	7.4 (7.4, 7.4)	7.4	7.4 (0.3, 7.4)	0.44	7.4	7.4 (7.4, 7.4)	< 0.0001	7.4	7.4 (7.3, 7.4)	0.001
pO2 (kPa)	4.8	4.8 (4.6, 4.9)	4.8	4.8 (4.6, 5.0)	0.79	5.1	5.1 (4.9, 5.3)	< 0.0001	4.9	4.9 (4.7, 5.1)	0.001
Bicarbonate (mEq/L)	24.6	24.8 (22.6, 26.5)	24.7	24.8 (22.5, 26.9)	0.78	25.8	25.8 (24.2, 27.4)	< 0.0001	25.5	25.5 (23.6, 27.2)	0.001
Lactate Dehydrogenase (LD) (IU/L)	291.9	237.3 (230.9, 250.0)	399.4	268.0 (230.3, 320.0)	0.08	272.4	235.7 (235.7, 235.7)	0.03	330.4	235.7 (226, 301)	0.29

* first 25 lab items or those with a false discovery rate-adjusted p-value< 0.1 are shown

**Fig. 3:**
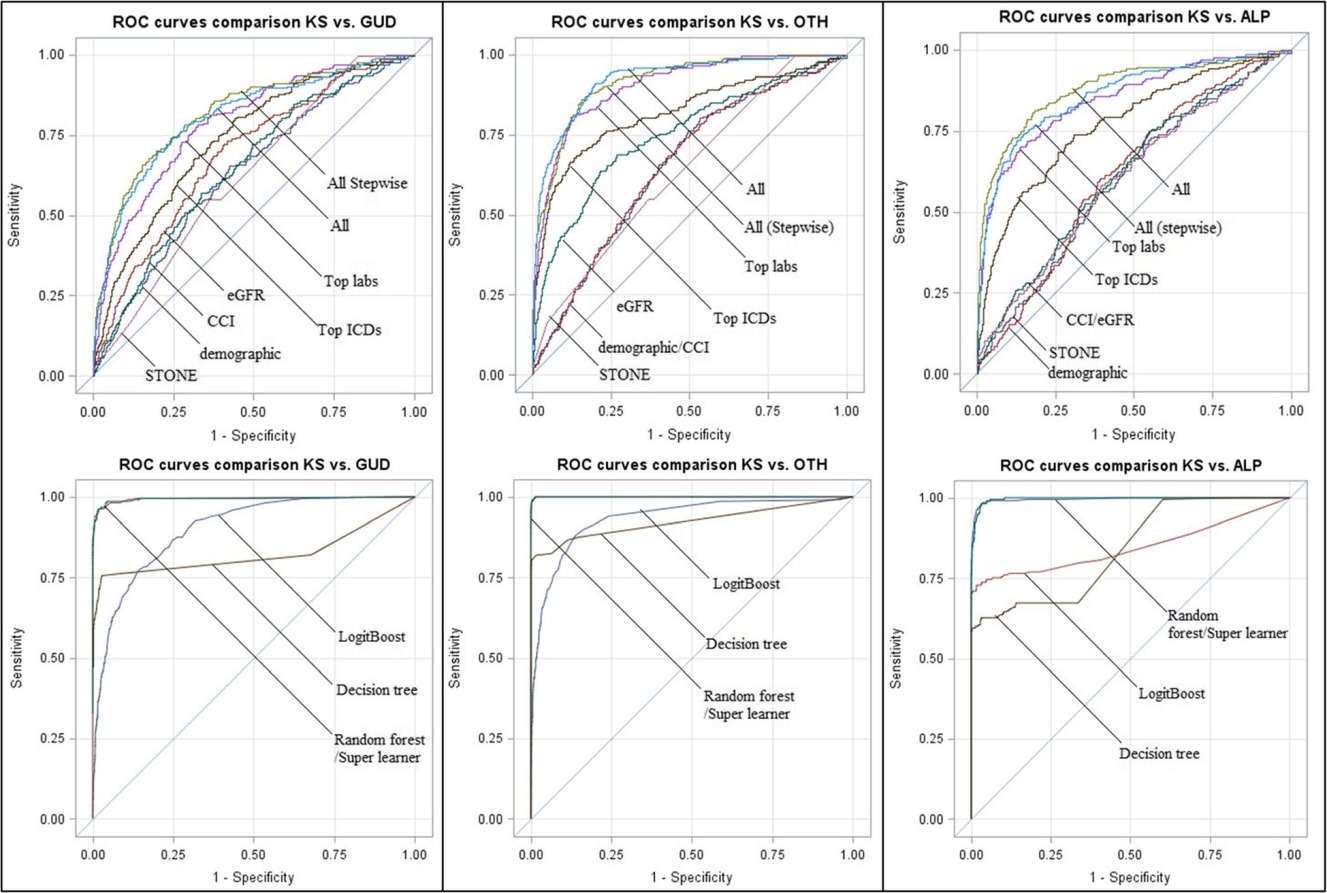
Model comparison via AUROC. Legend: Left panels: kidney stone (KS) formers vs. other genitourinary diseases (GUD); middle panels: KS vs. other non-genitourinary (OTH) conditions; right panels: KS vs. acute localized pain (ALP) in the abdomen, back, flank, or groin. Top panels: logistic regression models upon stepwise feature selection, fit on selected input domains; Bottom panels: comparison of machine learning techniques on the full input set. Curves shown are averaged over 10-fold cross-validation, i.e. using the test sets

In order to derive a multi-domain diagnostic model of KS diagnosis, we fitted different logistic models on selected covariate input domains, as specified in the Methods section, and compared against the STONE. Table 4 summarizes the performance indices for models (a) through (h), showing average (st.dev.) AUROC, sensitivity, specificity across 10-fold cross-validation runs (i.e. results obtained on the test data), along with the best Youden’s J. Figure 3 (top panels) shows the ROC curves for each model, also obtained by averaging the 10 tests sets, for the KS vs.)GUD, KS vs. OTH, and KS vs. ALP data samples. Overall, model (f), i.e. the top-ranked ICD-9 diagnosis and laboratory tests plus demographic variables, and model (g), i.e. the stepwise selection of features included in model (f), showed the best performance, with AUROCs ~ 80%. All other models were significantly less performant (adjusted *p* < 0.05) than these two. Following cross-validated AUROC ranking, the second best-performing models were those with top-ranked ICD-9 codes (d), laboratory tests (e), CCI (b), eGFR (c), and demographics alone (a).

**Table 4:** Comparison of prediction performance of different models, using 10-fold cross validation

Model/Outcome		AUC	Sensitivity	Specificity	J
kidney stones (KS) vs. other genitourinary diseases (GUD)					
(a)	Demographic	0.63 (0.02)	0.69	0.51	0.20
(b)	Charlson’s comorbidity index	0.69 (0.02)	0.69	0.62	0.31
(c)	eGFR	0.62 (0.02)	0.65	0.56	0.21
(d)	ICD	0.74 (0.02)	0.75	0.63	0.38
(e)	Labs	0.76 (0.02)	0.67	0.74	0.41
(f)	All	0.81 (0.02)	0.75	0.76	0.51
(g)	All (Stepwise)	0.80 (0.02)	0.76	0.71	0.47
(h)	STONE	0.62 (0.02)	0.55	0.64	0.19
KS vs. other conditions (OTH)					
(a)	Demographic	0.65 (0.02)	0.64	0.62	0.27
(b)	CCI	0.65 (0.02)	0.68	0.57	0.25
(c)	eGFR	0.71 (0.02)	0.59	0.75	0.35
(d)	ICD	0.82 (0.02)	0.68	0.87	0.55
(e)	Labs	0.90 (0.01)	0.81	0.87	0.68
(f)	All	0.92 (0.01)	0.90	0.80	0.70
(g)	All (Stepwise)	0.92 (0.01)	0.90	0.81	0.71
(h)	STONE	0.64 (0.02)	0.62	0.65	0.27
KS vs. acute localized pain (ALP)					
(a)	Demographic	0.60 (0.02)	0.71	0.47	0.18
(b)	Charlson’s comorbidity index	0.62 (0.02)	0.60	0.61	0.21
(c)	eGFR	0.57 (0.02)	0.59	0.44	0.15
(d)	ICD	0.77 (0.02)	0.74	0.69	0.43
(e)	Labs	0.85 (0.02)	0.66	0.90	0.56
(f)	All	0.88 (0.01)	0.78	0.85	0.63
(g)	All (Stepwise)	0.86 (0.01)	0.81	0.82	0.63
(h)	STONE	0.61 (0.02)	0.58	0.60	0.18

Notably, models using top-ranked ICD-9 diagnostic codes showed high sensitivity and moderate specificity, while models using top lab tests showed moderate sensitivity and high specificity, while both high sensitivity and high specificity were achieved in the multi-domain models. The STONE model (h) yielded relatively low AUROC (62% for KS vs. GUD, 64% for KS vs. OTH, and 61% for KS vs. ALP).

When we added the ICD-9 code for hematuria and other GUD codes to the set of input variables for models (f) and (g), performance increased significantly: For KS vs. GUD, model (g) achieved AUROC of 88% (*p* < 0.0001 w.r.t. models with non GUD-specific ICD-9 codes) with sensitivity of 77% and specificity of 87%; for KS vs. OTH, model (g) achieved AUROC of 98% (*p* < 0.0001), with sensitivity of 88% and specificity of 98%; for KS vs. ALP, model (g) achieved AUROC of 87% (*p* < 0.0001), with sensitivity of 81% and specificity of 82%. Model (f) had very similar performance (not shown). However, these GUD variables were measured concurrently with KS, so we did not include them in our final prediction model, but it could be used as input if these GUD variables happened in one’s history to improve the predictivity and performance of the models.

**Fig. 4:**
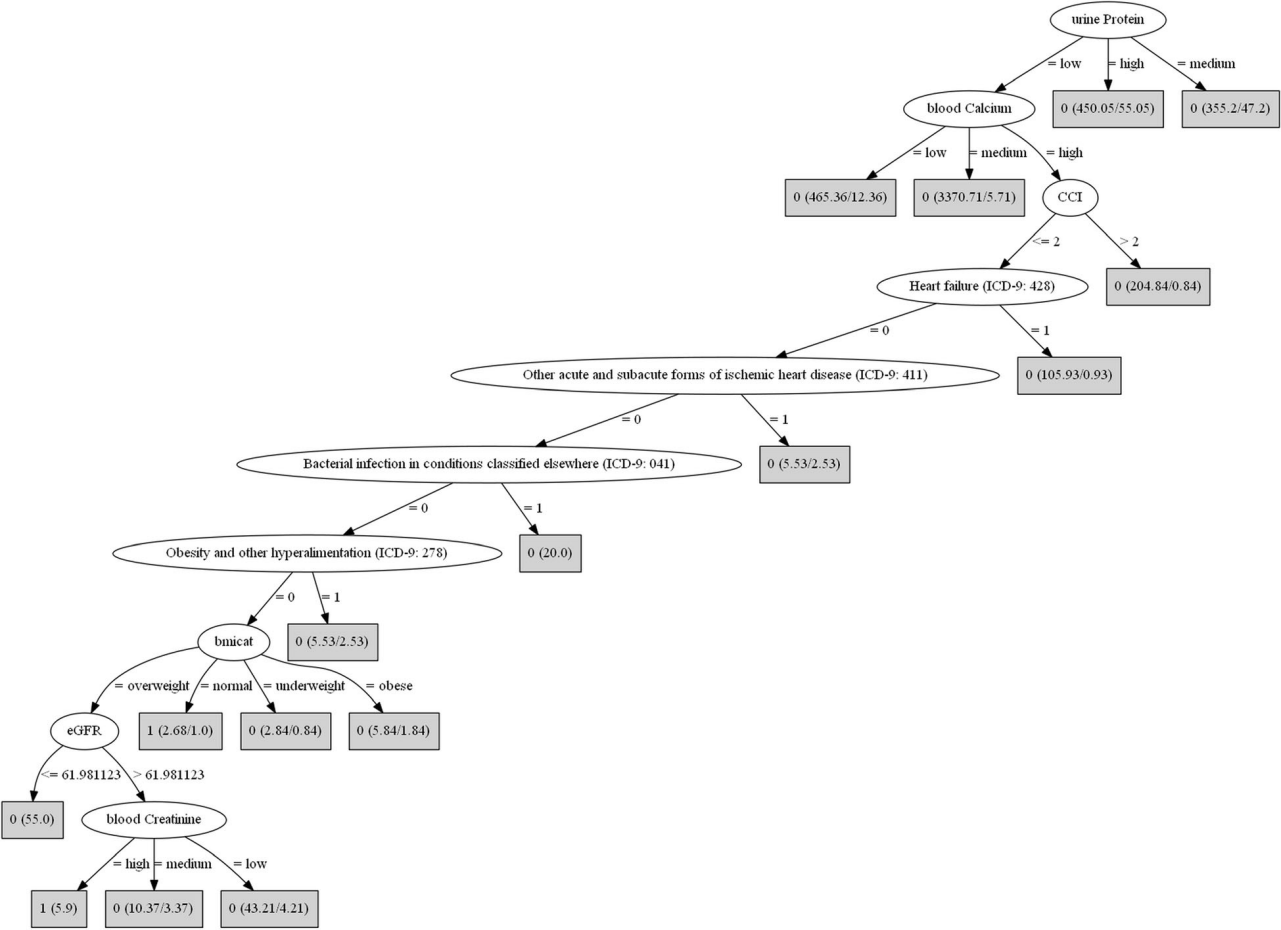
Decision tree for the diagnosis of KS patients vs. ALP patients Legend: Each leaf node contains the predicted class (1 if KS, 0 if ALP) and the numbers between parentheses indicate total number of instances (first number) reaching the leaf, and the number of those instances that are misclassified (second number).

When we applied the machine learning techniques, using the same cross-validation settings, for the comparison between KS and GUD or OTH, we did not observe a substantial increase in performance indices with the usage of the LogitBoost selector in alternative to the stepwise, but an increased performance was observed for KS vs. ALP (*p* < 0.0001). The variables selected by the LogitBoost were concordant with the variables selected from stepwise logistic regression model (g), although the LogitBoost tended to select a few more. The decision tree showed a peculiar behavior as compared to the logistic regression, with increased sensitivity at higher specificity but then lower plateau. The random forest showed higher (almost perfect) AUROC and sensitivity/specificity (significant below the 0.0001 level with respect to the logistic regression and decision tree) and the super learner was comparable to the random forest. In fact, the highest weight of the super learner was that of the random forest, followed by the decision tree, a single rule, and the LogitBoost. The bottom panels of Fig. 3 show the cross-validated ROC curves corresponding to KS vs. GUD, KS vs. OTH, and KS vs. ALP. The decision tree for KS vs. ALP is depicted in Fig. 4. Using the SMOTE, performance results for all models were lower but ranking similar (not shown).

The final model of choice was the stepwise-selected model (g), because in conjunction with optimal performance, it included fewer variables than model (f) (15 variables for each comparison vs. 50 variables in model (f)). Table 5 displays the final model (with odds ratios and confidence intervals) which we name as the Diagnostic Acute Care Algorithm for Kidney Stones (DACA-KS). The stepwise regression for KS vs. GUD yielded a few nonspecific predictors (e.g. nonspecific findings on examination of blood (ICD-9: 790), Other complications of procedures (ICD-9:998)) which were removed without loss in performance. In addition, although random forest and super learner showed better performance, given the high class imbalance we have in the sample population, we cannot sure about the generalizability of these models in different dataset, so we focused more on interpretability especially when the logistic regression model had good performance as well. In fact, the SMOTE performance estimates of the super learner as well as of the random forest are lower.

**Table 5:** The Diagnostic Acute Care Algorithm - Kidney Stones (DACA-KS)

Items	Kidney Stones vs. Other Genitourinary Diseases		Kidney Stones vs. Other Conditions		Kidney Stones vs. Acute Localized Pain	
	OR (95% CI)	*p*-value	OR (95% CI)	*p*-value	OR (95% CI)	*p*-value
Insurance	1.70 (1.25, 2.32)	0.001				
Charlson Comorbidity Index	0.81 (0.75, 0.88)	< 0.0001	0.91 (0.84, 0.97)	0.01	0.88 (0.82, 0.94)	0.001
Certain adverse effects (ICD-9: 995)	2.93 (2.14, 4.00)	< 0.0001	6.33 (4.47, 8.96)	< 0.0001		
Hypertensive renal disease (ICD-9: 403)	0.55 (0.34, 0.89)	0.01	5.37 (2.96, 9.77)	< 0.0001		
Obesity and other hyperalimentation (ICD-9: 278)	2.06 (1.28, 3.32)	0.003			2.18 (1.25, 3.82)	0.04
Candidiasis (ICD-9: 112)	2.37 (1.39, 4.06)	0.002				
Decreased libido and other ill-defined conditions (ICD-9: 799)	2.30 (1.35, 3.93)	0.002	1.98 (1.08, 3.64)	0.03	3.40 (1.85, 6.27)	0.001
urine white blood cells (WBC)	1.13 (1.10, 1.16)	< 0.0001	1.26 (1.22, 1.30)	< 0.0001	1.15 (1.11, 1.19)	< 0.0001
urine pH	1.45 (1.20, 1.74)	< 0.0001				
urine protein	1.26 (1.01, 1.57)	0.04	2.19 (1.78, 2.69)	< 0.0001	1.99 (1.61, 2.45)	< 0.0001
blood Magnesium	0.17 (0.08, 0.35)	< 0.0001				
blood Chloride	3.97 (2.06, 7.65)	< 0.0001	5.05 (2.02, 12.59)	0.001	2.67 (1.01, 7.03)	0.05
blood Albumin	1.78 (1.37, 2.32)	< 0.0001				
blood red blood cells (RBC)	1.60 (1.22, 2.10)	0.001	1.49 (1.11, 1.99)	0.01	1.77 (1.24, 2.52)	0.002
Disorders of fluid, electrolyte, and acid-base balance (ICD-9: 276)			1.70 (1.23, 2.37)	0.002		
Other bacteria infections (ICD-9: 041)			5.47 (3.60, 8.31)	< 0.0001	2.67 (1.81, 3.93)	0.01
Sleep disorders (ICD-9: 327)			3.06 (1.76, 5.31)	< 0.0001	3.29 (1.80, 6.02)	0.001
Chronic pulmonary heart disease (ICD-9: 416)			2.07 (1.13, 3.79)	0.02		
Acute and subacute necrosis of liver (ICD-9: 570)			3.14 (1.38, 7.14)	0.01		
blood pO_2_			0.52 (0.36, 0.76)	0.001		
Neurotic disorders (ICD-9: 300)					2.28 (1.34, 3.88)	0.03
Hyperplasia of prostate (ICD-9: 600)					2.88 (1.48, 5.60)	0.02
blood CO2					0.70 (0.61, 0.80)	< 0.0001
blood phosphate					0.60 (0.47, 0.76)	< 0.0001
urine bands					1.15 (1.07, 1.24)	0.002
urine RDW					0.79 (0.71, 0.88)	< 0.0001

## Discussion

In this large sample of individuals admitted to acute care between 2000 and 2012, we aimed to infer a multi-domain, personalized, diagnostic algorithm risk assessment for KS disease. With a robust model collection and selection framework, under cross-validation settings, we demonstrated that the integrated model improves both specificity and sensitivity as compared to a single domain model. Also, it includes more extensive parameters compared to the STONE score. The STONE score utilizes presentations of KS-related symptoms (pain, hematuria, nausea/vomiting) and two demographic predictors (gender and race). In our sample population, only a small proportion of (KS) patients had hematuria and nausea/vomiting present or recorded. Our study evaluated thousands of potential predictors among the different domains, comparing relative proportions and shifts in distributions between KS formers and the GUD, OTH and ALP groups, our model can make personalized prediction for each individual based on his/her parameters from different domains. The features used in our final models are usually routinely tested in critical care unit, or tested at admission, therefore, all information to implement our model should be available in an ICU setting, and can be easily adapted to different clinical settings by adding or removing features. We report a series of novel findings in KS that are significantly different than GUD, OTH and ALP populations and which could aid in the triage of patients when they present to the ED or are admitted/transferred into critical care. A number of these variables are worth of discussion in detail.

In our study cohort, we found that KS peaked at the 7th decade of age; with variation of prevalence at different age groups between both genders, overall, we found a higher prevalence of females in this cohort. KS prevalence was the highest in non-Hispanic whites, similarly to other studies [1]. Lower rates of private insurance coverage were found in KS (comparing with OTH), which suggests that socio-economic status may contribute to risk factors associated with KS. Previous studies showed that lower income [1] and lower coverage of private insurance [24] are associated with higher risk of KS [25].

In our population, KS formers had the highest prevalence of obesity when compared to the GUD/OTH/ALP groups, and our final multivariate model suggested that patients with obesity are two times more likely to be diagnosed with KS comparing with GUD or ALP patients.

We found that KS, OTH and ALP were a healthier cohort with lower CCI and higher eGFR when compared to the GUD. Previous studies have demonstrated that KS formers have higher risk of developing chronic kidney diseases [4, 26]; in fact, in our study we found a tendency to a decreased eGFR in KS with respect to OTH/ALP groups, and this points to the necessity of monitoring and management of KS to prevent progression into chronic kidney disease.

The most common diagnosis associated with ED visits was hypertension, and its prevalence was higher in patients with KS comparing to GUD and ALP. Disorders of fluids, electrolytes, and acid-base balance was also frequently found in KS and GUD, but not in the diagnosis in the OTH/ALP group. A meta-analysis found that increasing water intake was associated with significantly reduced risk of kidney stones and it was dose dependent for each increase of 500 ml of water [27]. For KS formers, the single most significant preventive measure is increasing fluid intake. In the GUD population, disorders of fluids and electrolytes are a well-known entity. In addition, diseases of acid base and electrolytes such as renal tubular acidosis (RTA) and partial RTA, which may present with hyperchloremic acidosis, hypokalemia, and normal or minimally reduced GFR [28], also have a higher prevalence of KS [29]. Interestingly, in our KS cohort we found higher levels of serum chloride and lower levels of serum bicarbonate, lower serum potassium levels, and elevated urine protein comparing to the GUD/OTH/ALP groups, Additional research efforts may be able to fully elucidate the significance of these findings.

We found that purpura and other hemorrhagic conditions were higher in the KS population when compared to the OTH/ALP population but there was no significant difference when compared to the GUD group.

The distribution of serum lipase and creatinine kinase MB isoenzyme were significantly lower in KS as compared to the GUD and OTH/ALP groups. Renal handling of lipase involves removal of lipase from serum by glomerular filtration of lipase with nearly complete absorption of free oxalate in the bowel lumen [30]. Disorders of lipid metabolism have been associated with the metabolic syndrome and obesity [31]. Lower levels of lipase in the KS group needs to be further elucidated as there have not been previous reports of this finding. Creatinine kinase MB (CK-MB) is an enzyme that is elevated in renal disease and it may be elevated even in the absence of myocardial injury; however, the significance of its elevation is controversial [32]. Further investigation is warranted to unveil both the role of low lipase and CK-MB isoenzyme in KS formers.

A set of neurologic findings in our study demonstrated that migraine headaches were higher in KS and OTH compared to GUD/ALP. Sleep disorder, neurotic disorder, and depression disorder were also higher in KS patients. Migraine headache medications such as Topamax promote an (RTA)-like phenomenon [33]. Sleep disorders and fatigue have been associated with migraine headaches [34]. In our analysis, sleep disturbances and low libido were correlated with the diagnosis of KS when compared to GUD, OTH and ALP. Low libido due to low testosterone could be correlated with poor sleep quality, since a normal circadian rhythm/cycle is necessary for central effects on normal testosterone production [35]. Low testosterone levels not only associated with low libido but also have been related with KS, Otunctermur et al. showed that male KS patients had lower testosterone levels, although the potential causal relationship were not confirmed [36].

Perhaps the most important finding and among high morbidity and mortality conditions, septicemia and candidiasis were found to have a high correlation with KS formers only. Reyner et al. [37] reported that of patients presenting to the ED with urosepsis, one-tenth presented with anatomic urinary obstruction, and that mortality was higher in this group, occurring in almost one-third of cases. Early imaging is suggested in this group of patients, due to suspected anatomic obstruction and need for immediate intervention to avoid mortality. Our data confirms this finding of a higher rate of urosepsis in KS patients when compared to other groups. This suggests that, as part of an algorithm to identify patients with KS, a high index of suspicion should trigger immediate action with early imaging to identify anatomic urinary obstruction in septic patients to prevent mortalities. In addition, the presence of candidiasis was found to have a higher association a KS diagnosis. Candidiasis is a fungal infection that can vary in presentation-from local to systemic and invasive, it may be found among debilitated, elderly and inpatients with indwelling urethral catheters [38], combining with our findings, patients presenting to the ED with candiduria may be considered for immediate imaging to identify any potential anatomic obstruction of the urinary tract. Interestingly, some variables in the model were not directly associated with risk of kidney stone: comparing to OTH patients, KS patients were more likely to have chronic pulmonary heart disease or acute and subacute necrosis of liver. These conditions might be associated with certain KS prognostic outcomes. Future studies the help further the understandings of these associations are needed.

There are several limitations of our study. First, we analyzed a sample from a single site, without external validation; the characteristics of patients in the KS, GUD, OTH and ALP are different and there may be a selection bias which we did not adjust for. In addition, many potentially useful lab tests were dropped because of low frequency in the KS group; other relevant lab predictors for KS may be found outside those routinely measured in people being triaged at the ED based on admission’s symptoms. Second, there was a high-class imbalance, for which the power of the study can be affected, as well as the derivation of a diagnostic model, even though we tried to address in part this issue using the SMOTE technique. Third, when using logistic regression, we did not consider interactions among variables (considering only two-ways interactions would have produced n^2^ variables, and we would have needed to use more efficient libraries, with parallel or cloud computing), therefore the model assumed a linear relationship. Ensemble methods, i.e. the random forest and the super learner, achieved almost perfect performance, but the result was not confirmed with the SMOTE class rebalancing, and this warrants further external validation using the TRIPOD protocol [39]. Even though we used nested cross-validation for parameter optimization, there may have been overfitting. Fourth, we acknowledge a subpar calculation of the STONE score because we could not assess the duration of pain, and the small number of subjects with vomiting and nausea in our sample indicating there may be under-reporting during data collection. Due to the cross-sectional nature of this study, we cannot determine the causality of the predictors for KS formation, but even using longitudinal database with variables only from earlier data, the causality of the predictors is still unable to be confirmed. Future studies may help address these limitations and help designing early-risk diagnostic models applicable to the general population.

Despite these limitations, our study provided a compact and high-performance diagnostic model for diagnosis of KS.

## Conclusions

DACA-KS could be integrated into electronic health systems; the algorithm has the potential used as an effective tool to help nurses and healthcare personnel during triage or clinicians making a diagnosis, streamlining patients’ management in acute care. As we enter the era of precision medicine, we envision a family of DACA- models for many other conditions in addition to KS, derived in the same way from big integrated biomedical data bases.

## Abbreviations

ALP: Acute localized pain
AUROC: Area under the receiver operating characteristics
BMI: Body mass index
CCI: Charlson Comorbidity Index
DACA: Diagnostic acute care algorithm
ED: Emergency department
eGFR: Estimated glomerular filtration rate
GUD: Genitourinary diseases
KS: Kidney stones
OTH: Other conditions
SMOTE: Synthetic Minority Over-sampling Technique
